# Synthesis of Ultrasmall Single-Crystal Gold–Silver Alloy Nanotriangles and Their Application in Photothermal Therapy

**DOI:** 10.3390/nano11040912

**Published:** 2021-04-03

**Authors:** Mirko Maturi, Erica Locatelli, Letizia Sambri, Silvia Tortorella, Sašo Šturm, Nina Kostevšek, Mauro Comes Franchini

**Affiliations:** 1 Department of Industrial Chemistry Toso Montanari, University of Bologna, Viale Risorgimento 4, 40136 Bologna, Italy; mirko.maturi2@unibo.it (M.M.); erica.locatelli2@unibo.it (E.L.); letizia.sambri@unibo.it (L.S.); silvia.tortorella2@unibo.it (S.T.); 2 Department for Nanostructured Materials, Jožef Stefan Institute, Jamova 39, 1000 Ljubljana, Slovenia; saso.sturm@ijs.si

**Keywords:** gold–silver nanotriangles, seed-mediated growth, surface coating, photothermal therapy

## Abstract

Photothermal therapy has always been a very attractive anti-cancer strategy, drawing a lot of attention thanks to its excellent performance as a non-invasive and pretty safe technique. Lately, nanostructures have become the main characters of the play of cancer therapy due to their ability to absorb near-infrared radiation and efficient light-to-heat conversion. Here we present the synthesis of polyethylene glycol (PEG)-stabilized hybrid ultrasmall (<20 nm) gold–silver nanotriangles (AuAgNTrs) and their application in photothermal therapy. The obtained AuAgNTrs were deeply investigated using high-resolution transmission electron microscopy (HR-TEM). The cell viability assay was performed on U-87 glioblastoma multiforme cell model. Excellent photothermal performance of AuAgNTrs upon irradiation with NIR laser was demonstrated in suspension and in vitro, with >80% cell viability decrease already after 10 min laser irradiation with a laser power *P* = 3W/cm^2^ that was proved to be harmless to the control cells. Moreover, a previous cell viability test had shown that the nanoparticles themselves were reasonably biocompatible: without irradiation cell viability remained high. Herein, we show that our hybrid AuAgNTrs exhibit very exciting potential as nanostructures for hyperthermia cancer therapy, mostly due to their easy synthesis protocol, excellent cell compatibility and promising photothermal features.

## 1. Introduction

Noble metal nanoparticles have received great attention due to their peculiar optical properties, which allow for unique applications in a huge number of fields. Such properties derived from the phenomenon of the so-called localized surface plasmon resonance (LSPR), which is particularly relevant for gold nanoparticles (AuNPs) [1]. LSPR strongly depends on the size and shape of the nanoparticles; thus, research had seen the arising of a multitude of geometrically different gold nanoparticles: nanospheres, nanorods, nanotriangles, nanostars, and variously-branched nanoparticles have been synthesized and investigated for opening up novel fields of application [2,3].

Among other, gold nanotriangles (AuNTrs) stand out for unique optical and plasmonic properties, such as very large absorption and scattering cross-sections close to the near-infrared (NIR) region, which allow them to be one of the best candidates as heater-nanoparticle in hyperthermia cancer treatment [4,5]. Despite these features, AuNTrs are actually less investigated in comparison to gold nanorods, due to the high complexity of available synthetic protocols and poor yields [6]. Moreover, the particles obtained with nowadays procedures show big lateral dimensions (average edge length of about 284 nm [7]) and no AuNTrs with size below 50 nm could be found in the literature. In addition, the stability of these NPs is low, and oxidation with subsequent reshaping phenomena may occur a few hours after synthesis if no proper surface coating is developed and exploited [8]. The absorption spectrum for AuNTrs has a band located in the NIR range and represents in-plane dipole resonance. However, most likely an increase of the NTs edge length induces a maximum redshift; thus, most of the particles reported in literature present an absorption maximum wavelength in the 900–1000 nm range [9], which may limit the lasers available for hyperthermia treatment. Indeed, the optimal window for tissue irradiation is generally recognized to be between 700 and 800 nm (the so-called first biological window), where the absorption of tissues and hemoglobin is at a minimum [10].

In recent literature, it has been demonstrated that control of size, and in certain cases of shape [11], of gold nanoparticles could be achieved by the addition of a small amount of silver seeds into the growth solution, where gold is reducing and forming nanoparticles. In particular, gold nanorods are synthesized in presence of silver nitrate and the control of lateral dimensions of the obtained rods should be attributed to silver ions: the higher the amount of silver the smaller the length of obtained rods [12]. Size, shape, and optical properties of the obtained nanosystems may vary depending on the technique employed for their synthesis. Recently, AuAg core-shell and hollow nanoparticles have been prepared and discussed in terms of their photothermal properties [13,14,15], but recently published numerical simulations suggest that AuAg alloy nanosystems are characterized by sharper and more intense absorption properties compared to core-shell structures [16]. Moreover, the ratio between gold and silver atoms seems to play a role in determining the position of the LSPR peak [17].

Following this idea, we investigated the possibility to reduce NTrs lateral dimension by adding silver during the growth of the nanoparticles, thus limiting the enlargement, and obtaining an alloy of Au-Ag. To the best of our knowledge, no bimetallic Au-Ag nanotriangles (AuAgNTrs) were prepared up to now. In this paper we present an easy, reliable, and mild-conditions synthesis of AuAgNTrs stabilized with polyethylene glycol (PEG) onto the surface. The properties of the obtained NTrs were deeply investigated in order to confirm their similarity with the ones of pure AuNTrs. After physical–chemical properties examination, we also demonstrate the possibility to use AuAgNTrs as a heater for hyperthermia therapy during laser irradiation.

## 2. Materials and Methods

All chemicals were purchased from Sigma-Aldrich (St. Louis, MO, USA) and used as received. Purity of chemicals was >97% for BDAC, >99% for AgNO_3_, 99.995% for HAuCl_4_, 99% for NaBH_4_, and >99% for ascorbic acid. Gold and silver standard solutions at 1, 2, 5, and 10 mg/mL for atomic absorption spectroscopy were prepared by diluting in 30% aqua regia the appropriate amounts of 1000 mg/mL TraceCERT^®^ solutions. Polyethylene glycol with thiol, amino, methoxy and carboxylic acid end groups (HS-PEG-COOH, MW~3 kDa, HS-PEG-NH_2_, MW~3 kDa, HS-PEG-OMe, MW~3 kDa) were purchased from Rapp Polymere GmbH (Tübingen, Germany). All aqueous solutions were prepared with deionized water (ultrafiltration system Milli-Q, Millipore with a measured resistivity above 18 MΩ/cm). Malvern (Malvern, UK) Zetasizer-Nano-S with a 532 nm laser beam was used for dynamic light scattering (DLS) measurements. ζ-potential measurements were performed in DTS1060C-Clear disposable zeta cells at 25 °C. Atomic absorption spectroscopy (AAS) analyses were conducted on SpectraAA 100 Varian (Agilent, Santa Clara, CA, USA). Gravimetric analysis was performed to determine the final concentration of the suspensions by drying 100 μL of solution at 120 °C for 24 h and then accurately weighting the residual amount of the dry matter. Samples for the transmission electron microscopy (TEM) analysis were prepared by adding a drop of nanoparticles suspension on a lacey carbon coated TEM grid (Ted Pella, Inc., Redding, CA, USA). The selected-area electron-diffraction (SAED) analysis combined with the conventional transmission electron microscopy (TEM) and high-angle annular dark-field scanning transmission electron microscopy (HAADF-STEM) imaging was performed by applying a Cs-aberration-corrected probe (JEM-ARM 200CF; JEOL, Tokyo, Japan) using the cold field emission source operated at 200 keV, with a spatial resolution in STEM imaging mode of 0.1 nm. The probe convergence semi angle and the collection semi angle for the HAADF detector were set to 24 and between 45 and 180 mrad, respectively. The energy dispersive X-ray spectrum (EDXS) images were performed by using the EDXS (Centurio 100 mm^2^, JEOL, Tokyo, Japan) system under continuous scanning mode with a pixel dwell time of 25 microseconds and a probe current of 250 pA, therefore degrading the spatial resolution to approaching values of 0.2 nm.

Photothermal experiments were performed using an FC-808 Fiber Coupled Laser System (CNI Optoelectronics Tech, Changchun, China) in the continuous-wave operation mode at 808 nm with different laser power. Optical lens was used to focus the continuous-wave diode light on a quartz cuvette (size: 1 × 1 × 3 cm) with a spot size of 8 mm. Control (DI water) and NPs suspension with 4 different concentrations (8.8–70 μg/mL) were irradiated with NIR laser (λ = 808 nm) at *P* = 1, 2, and 3 W/cm^2^ for 5 min. The temperature of the suspension (volume = 1 mL) was measured with a J-type Teflon thermocouple, immersed in the cuvette, and connected to a computer for a real-time data collection. Photothermal experiments on cells were performed in 24-well plates (5000 cells/well) after 24 h incubation with control or NPs suspension with 5 and 50 μg AuAg/mL. Each well was irradiated for 10 min with *P* = 3 W/cm^2^. Control samples were irradiated with *P* = 1, 3, and 5 W/cm^2^ to evaluate the effect of laser power on cell viability. Each well contained 0.2 mL of NPs in cell culture medium. During irradiation, the temperature of irradiated wells was monitored using a thermal camera (FLIR E8, FLIR Systems, Inc., Victoria, Canada). Cell viability assay and optical inspection (inverted fluorescence microscope Olympus IX81, Olympus Corporation, Tokyo, Japan) of cells were performed 24 h post-treatment to observe any delayed effect of treatment on the cells.

### 2.1. Synthesis of Ultrasmall AuAg Nanotriangles (AuAgNTrs)

Firstly, silver seeds have been prepared by dissolving 188 mg of benzyl cetyl dimethyl ammonium chloride (BDAC) in 4.93 mL of water in a glass vial by gentle heating and magnetic stirring. Then, 20 μL of 100 mM AgNO_3_ solution were added, and the obtained solution was let to equilibrate at 30 °C in a water bath. The vial was then wrapped in aluminum foil to protect it from light and 600 μL of 10 mM NaBH_4_ were added to start the nucleation of Ag seeds. The solution has been stirred for a few seconds, then seeds were allowed to age for 2 h at 30 °C in the dark. The growth solution was then prepared by dissolving 1.98 g of BDAC in 50 mL of water in a 100-mL round-bottomed flask by gentle heating and magnetic stirring. The solution was then placed in a water bath at 30 °C and 260 μL of 100 mM AgNO_3_ and 210 μL of 100 mM HAuCl_4_ were sequentially added. Then, 1.34 mL of 100 mM ascorbic acid have been added as the reducing agent, and the solution turned colorless due to the reduction of Au(III) ions to Au(I) ions. After ageing the seeds for 2 h, 750 μL of their solution were added to the growth solution, stirred for 30 s then let to sit at 30 °C for 1 h. Afterwards, the solution has been centrifugated (20 min, 6 krpm) to remove co-formed nanowires and the blue supernatant containing BDAC-coated nanotriangles has been concentrated to 10 mL.

### 2.2. Synthesis of PEG-Coated Ultrasmall AuAg Nanotriangles (AuAgNTrs@PEG)

Stabilization of the nanostructure was performed by dropping the nanotriangles in a solution made of 50 mg of HS-PEG-COOH, 50 mg of HS-PEG-NH_2_ and 50 mg of HS-PEG-OMe in 10 mL of ethanol. The obtained mixture was then magnetically stirred overnight at room temperature, and purification has been performed on centrifugal membranes (MWCO 100 kDa, 3 krpm) and repeated washes with ethanol (3×) and then with water (3×). The final sample is then collected and diluted to 10 mL with water and then stored at +4 °C.

### 2.3. Cell Viability Tests

The experiments with U-87 cells (glioblastoma multiforme) were approved by the Veterinary Administration of the Slovenian Ministry of Agriculture and Forestry in compliance with the Animal Health Protection Act and the Instructions for Granting Permits for Animal Experimentation for Scientific Purposes. U-87 cells were obtained from ATCC (LGC Standards, Germany). Cells were seeded with a density of 5000 cells/well on 24-well plates (Corning Costar, TC-Treated). Cells were incubated for 24 h (at 37 °C, 5% CO_2_ atmosphere) with NPs suspensions in cell culture medium (MEM from Gibco/Invitrogen + 10% fetal bovine serum (HyClone) + 100 units/mL penicillin + 100 μg/mL streptomycin) in the concentration range: 0–70 µg AuAg/mL (*V* = 0.2 mL per well, *n* = 3, for control *n* = 7). After incubation, cells were washed to remove any non-internalized NPs. The cell viability was determined using the colorimetric MTT assay with a 3-(4,5-dimethylthiazol-2-yl)-2,5-diphenyltetrazolium bromide (MTT) staining. The absorbance was measured at 570 nm using a Safire plate reader. For photothermal experiments, the same seeding and incubation procedure was applied with control or NPs’ suspension with concentration 5 and 50 μg AuAg/mL (*n* = 2). Cell viability assay (MTT) and optical inspection of cells were performed 24 h post photothermal treatment to observe any delayed effect of treatment on the cells. One-way ANOVA and Student’s *t*-test was used for our statistical analysis. The data were presented as mean ±SD for all experiments.

## 3. Results and Discussion

**Scheme 1:**
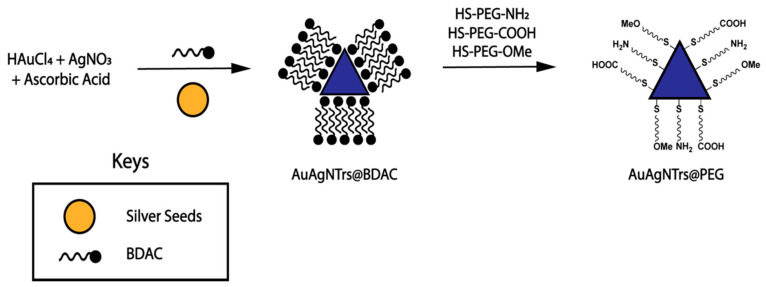
Schematic representation of the synthesis and surface functionalization of ultrasmall AuAg nanotriangles.

The reported seed-mediated synthesis procedure led to the simultaneous formation of ultrasmall AuAg nanotriangles and thin AuAg nanowires, in contrast with what reported by Qian et al. in 2014 [18], which developed a similar procedure for the formation of either Au nanowires or Au nanotriangles with an edge length of size of 25, 35, and 45 nm, depending on the reaction conditions. Moreover, in their study, they report the obtained nanotriangles are mostly composed by gold atoms, while silver atoms do not exceed 5 mol%. We believe that this difference could be related to the purity of the employed reactants, which has widely demonstrated to be a crucial parameter in determining the outcome of diluted seed-mediated approaches in the synthesis of noble metal nanostructures such as gold nanorods [19,20]. We observed that such small triangles undergo spontaneous reshaping at room temperature, leading to the obtainment of spherical particles, a phenomenon that can be easily detected by looking at the colour of the solution, which rapidly changes from blue to violet/purple: this is probably due to the high free energy of the edges of the triangular geometry. Due to this reason, a stabilization of the nanostructure with thiolated compounds is required to preserve its shape. Stabilization was performed with thiolated-PEGs: the thiol group at one end of the PEGs allows for anchoring to the AuAg surface while the other ending groups (amino, carboxylic and methoxy) guarantee effective stabilization in both slightly acidic and slightly basic pH (Scheme 1). Ethanol is added to disrupt BDAC micelles, allowing for the complete removal of the cationic surfactant from the nanotriangles dispersion [21].

**Figure 1:**
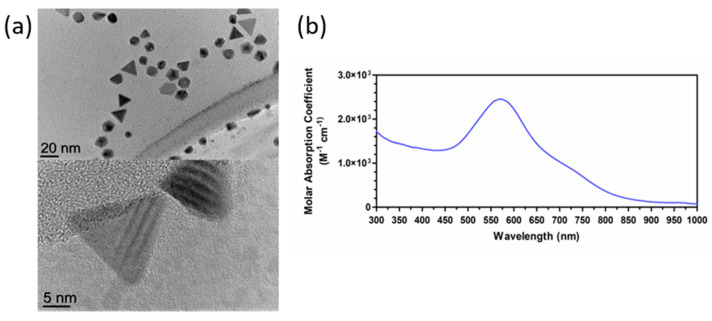
(**a**) Transmission electron microscopy (TEM) images of the obtained AuAgNTrs@PEG. (**b**) UV–Vis spectrum of AuAgN-Trs@PEG.

After purification, AuAgNTrs have been fully characterized. DLS analysis showed a hydrodynamic diameter of 43.1 ± 1.0 nm with a polydispersity index (PDI) of 0.274, thus confirming the obtainment of ultrasmall nanoparticles, and a surface ζ potential of −30.6 mV as expected for this kind of PEGylated coating: indeed the -COOH terminal group of PEG is deprotonated at neutral pH, leading to an overall negative surface charge. TEM images confirm the triangular geometry of the nanoparticles, with the presence of a minor amount of little spherical, pentagonal, and hexagonal impurities. The average edge length of the nanotriangles appears to be 17.5 ± 1.5 nm (calculated averaging *n* = 30 measured NTrs by TEM). TEM images also reveal that the nanotriangles display a perfectly ordered crystal structure, with aligned atom columns from one edge to the other (Figure 1).

**Figure 2:**
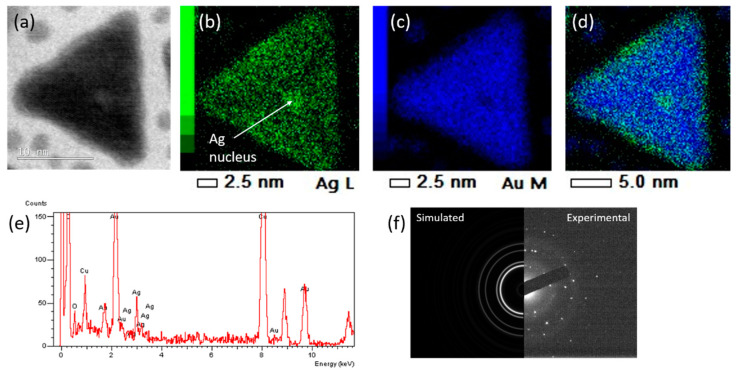
(**a**) TEM image of AuAgNTrs@PEG. Energy dispersive X-ray spectrum (EDXS) mapping of (**b**) Ag and (**c**) Au signal. (**d**) Overlap image composed of the Ag and Au EDXS signal. (**e**) Representative EDXS spectrum (Cu peaks belong to TEM grid) and (**f**) selected-area electron-diffraction (SAED) pattern analysis for observed nanotriangles compared to the simulated reflections for the Au face-centered cubic (FCC) crystal structures.

In order to determine the amount of gold and silver in the final suspension, we performed AAS analysis that revealed a prevalence of gold with a concentration of 0.52 mM in comparison to silver (0.32 mM) with a molar ratio of 38% Ag and 62% of Au. To better understand if the obtained suspension is composed of gold–silver alloy nanotriangles instead of being a mixture of pure silver nanotriangles and pure gold nanotriangles we performed EDXS mapping analysis (Figure 2) that showed the presence of both silver (green spots in Figure 2b–d) and gold (blue spots in Figure 2c,d) in the same nanotriangle, thus confirming the formation of gold-silver alloy. Moreover, the overlap of the EDS mapping (Figure 2d) clearly shows the presence of Ag nuclei in the core region of the triangles NPs. Quantitative EDXS analysis confirmed the above reported (Figure 2e) observations as well. According to six recorded spectra, the composition is Au_70±5_Ag_30±5_ (at.%).

An experimental SAED pattern analysis of the characterized nanotriangles demonstrates a good match with the simulated SAED pattern calculated from the face-centered cubic (FCC) Au crystal structures (Figure 2f). In general, solution-prepared Au and Ag triangular nanoprisms are single crystalline structures with FCC lattice parameters [22]. Dry mass was estimated to be 0.45 mg/mL, mainly attributed to the PEG coating. Finally, we determined the optical properties of the obtained particles by UV-Vis analysis and observed a single broad peak with a λ_max_ of 578 nm and a shoulder at 730 nm (Figure 1), which is expected due to the smaller dimensions of our nanotriangles in comparison to others reported in the literature so far [23].

**Figure 3:**
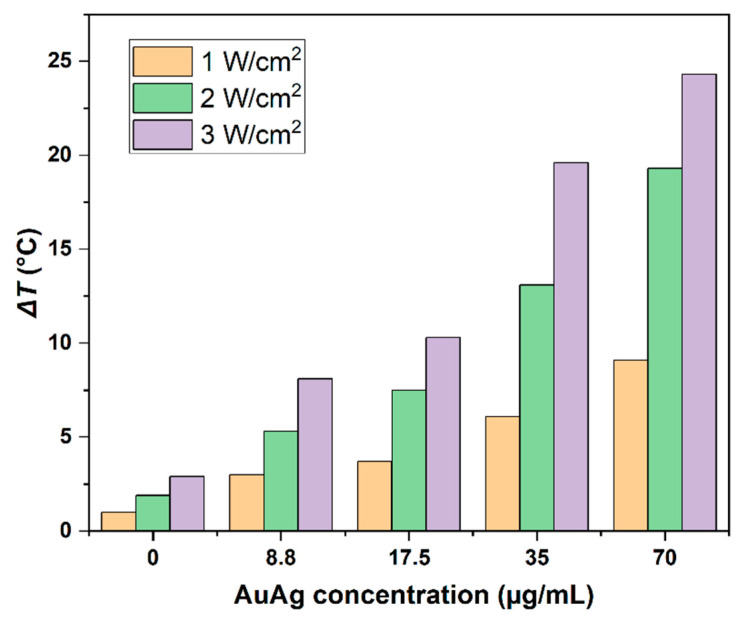
Δ*T* induced after irradiation for 5 min at different laser intensity (*P* = 1, 2, and 3 W/cm^2^) and AuAgNTrs concentration.

The photothermal properties of the AuAgNTrs in aqueous suspensions were evaluated in order to provide an initial indication of the photothermal performance. Nanoparticles suspension with 4 different concentrations (8.8–70 μg AuAg/mL) were irradiated with NIR laser (λ = 808 nm) at *P* = 1, 2, and 3 W/cm^2^ (sample volume 1 mL) for 5 min. Figure 3 shows the correlation between different nanoparticles concentration and different irradiation conditions (laser power) upon temperature variation. Expectedly, the highest concentration of AuAgNTrs (70 μg/mL) irradiated with 3 W/cm^2^ causes the highest temperature increase (Δ*T* = 24 °C). Since for photothermal therapy, Δ*T* of only 5–10 °C is generally requested, this requirement can be met with much lower nanoparticles concentration (<20 μg/mL at *P* = 3 W/cm^2^). Since no reports can be found in the literature about the photothermal efficacy of AuAgNTrs, we can only compare our results to AuNTrs. However, due to the different irradiation conditions (laser power and wavelength, different particle size, and sample concentration), the direct comparison is rather difficult. For example, Han et al. [24] prepared AuNTrs with an edge length of 94 nm and thicknesses of 10 nm, stabilized with HS-PEG-COOH and further functionalized with glucose, showing an absorption peak at 850 nm. Irradiation of suspensions (5–160 µg/mL of Au-PEG- glucose for 10 min) with 808 nm (*P* = 0.5 W/cm^2^) led to a maximum temperature increase of 20 °C. Next, Ma et al. [25] reported CTAC-capped AuNTrs with mPEG-SH coating (edge length 55 nm and thickness: 20 nm) and absorption peak at 669 nm. Laser irradiation with 650 nm-laser at *P* = 2 W/cm^2^ and nanoparticles concentration 5–40 µg/mL yield Δ*T*_max_ = 40 °C. Zhang et al. [26] prepared 110 nm AuNTrs, functionalized with HS-PEG-COOH with absorption peaks at 530 and 800–900 nm. Irradiation with 808 nm laser (*P* = 1 W/cm^2^, *t* = 750 s) and nanoparticles concentration 50 μg/mL resulted in Δ*T* =30 °C. All three articles reported AuNTrs with size > 50 nm and heating rates similar to our ultra-small AuAgNTrs. From this can be concluded that despite the small size, our AuAgNTrs are promising candidates for photo-thermal treatment.

**Figure 4:**
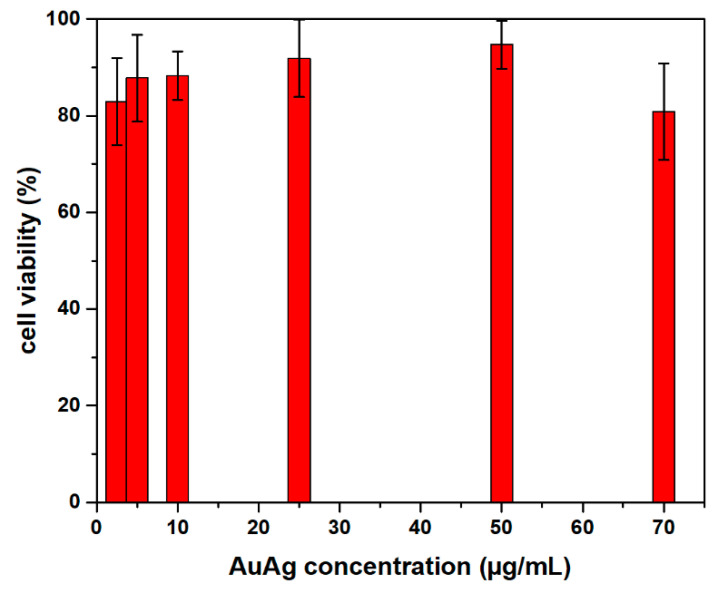
Cell viability (%) of U-87 cells determined using MTT assay after 24 h incubation with or AuAgNTrs in the concentration range 2.5–70 µg AuAg/mL (*n* = 3) normalized to the control that represents 100% viable cells. Data analysis revealed no statistically significant difference in cell viability values between tested groups (*p* > 0.05).

Then, cell viability assay on U-87 cells to focus the application of nanoparticles to glioblastoma multiforme was performed. After 24 h incubation, nanoparticles in the concentration range 0–70 µg AuAg/mL induced no significant cell viability drop (Figure 4), indicating good biocompatibility of AuAgNTrs under these testing conditions.

**Figure 5:**
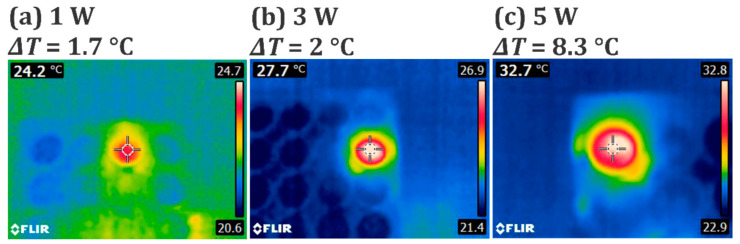
Thermal images of cells in 24 well plate. For each experiment, a single well was irradiated with an 808 nm laser for 10 min. Final temperatures and *ΔT* values (difference in the temperature before and after irradiation) values for cells irradiated with (**a**) *P* = 1, (**b**) *P* = 3, and (**c**) *P* = 5 W/cm^2^ are indicated.

Based on these promising results, in vitro photothermal experiment was performed. First, to test the influence of the laser power on the cell viability, cells without exposure to nanoparticles were irradiated with laser power *P* = 1, 3, and 5 W/cm^2^ for 10 min. During irradiation, the temperature was constantly monitored using a thermal camera (Figure 5). Due to the absence of nanoparticles, the temperature did not exceed 37 °C. However, and expectedly, higher laser power induced a higher temperature increase. From this can be concluded that a safe laser power exposure under these conditions is up to *P* = 3 W/cm^2^, where Δ*T* = 2 °C/10 min was observed, which is below the mild hyperthermia region (above 41 °C).

**Figure 6:**
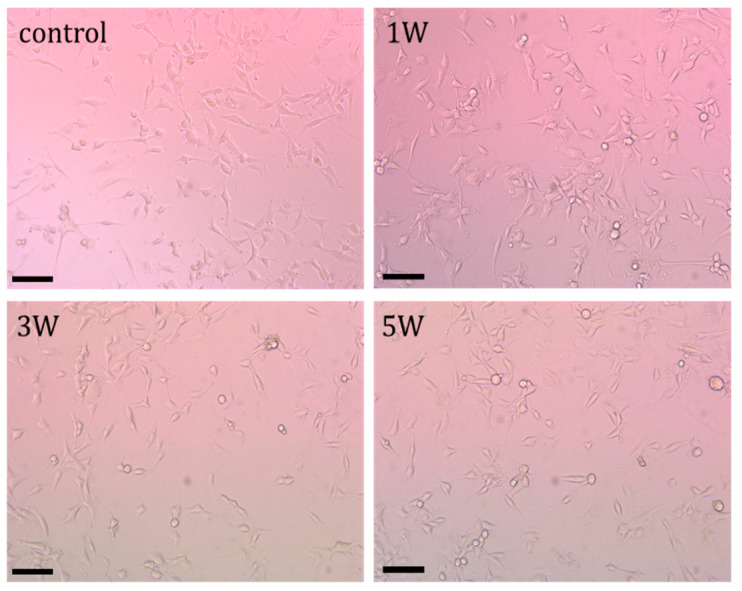
Optical microscopy images of cells 24 h post-irradiation with 808 nm laser for 10 min (*P* = 0, 1, 3 and 5 W/cm^2^). Scale bar 100 μm.

Cell viability assay and optical inspection of cells were performed 24 h post-irradiation to detect any possible delayed effect of the laser irradiation on the cells. Cell viability remained high in all cases (96 ± 2%, *n* = 3). To support these data, optical images are shown in Figure 6, indicating no significant morphological changes compared to a control sample. Therefore, to prevent the non-selective heating of the cells, the intermediate laser power (*P* = 3 W/cm^2^ with Δ*T* = 2 °C/10 min) was selected for the photothermal experiments with nanoparticles.

**Figure 7:**
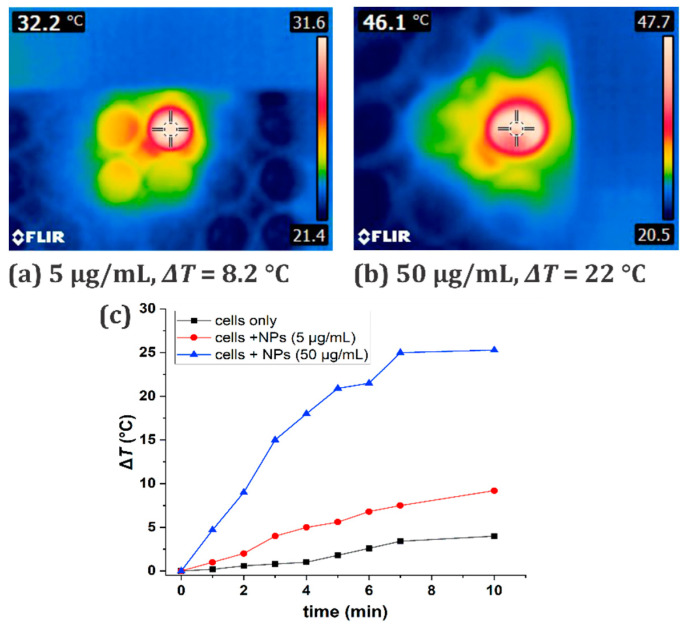
Thermal images of cells in a 24-well plate, irradiated with 808 nm laser for 10 min with *P* = 3 W/cm^2^, 24 h after incubation with nanoparticles (NPs) with concentration (**a**) 5 and (**b**) 50 μg AuAg/mL. (**c**) Temperature profile recorded during 10-min irradiation.

In the last step, cells were incubated for 24 h with AuAgNTrs with concentrations 5 and 50 μg/mL and then irradiated with laser (*P* = 3 W/cm^2^) for 10 min (*n* = 2). Thermal images with indicated final temperature and *ΔT* taken after 10-min irradiation of a single well are shown in Figure 7a (5 μg/mL) and 7b (50 μg/mL). The temperature profile recorded during 10-min irradiation is shown in Figure 7c, indicating a clear increase in temperature due to the presence of AuAgNTrs (8.2 and 25.3 °C for 5 and 50 μg AuAg/mL, respectively), compared to a control sample 2.5 °C, no NPs.

**Figure 8:**
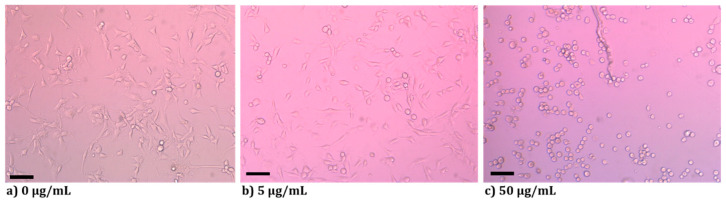
Optical images of cells 24 h post irradiation with 808 nm laser for 10 min (*P* = 3 W/cm^2^) after 24 h-incubation with AuAgNTrs with concentration (**a**) 0, (**b**) 5 and (**c**) 50 μg AuAg/mL. Scale bar 100 μm.

Finally, 24 h post-irradiation the cell viability dropped down to 20 ± 3% (*n* = 3) in the case of incubation and irradiation with 50 μg AuAg/mL (without irradiation cell viability remained high, Figure 4), while at lower concentration (5 μg AuAg/mL), cell viability decreased to 75 ± 3% (*n* = 3) compared to a control. Statistical analysis confirmed that the means of three populations (control, 5 μg AuAg/mL and 50 μg AuAg/mL) are not equal and the cell viability values between different groups differ significantly (*P* < 0.05). This is further supported with optical microscopy images (Figure 8), showing drastic morphological changes in cells exposed to higher NPs concentration due to the strong temperature increase compared to the control, where *ΔT* was minimal. In summary, in vitro photothermal experiments revealed that already a single cycle of laser irradiation successfully reduced the cell viability of cancer cells. This is in line with other best candidates for hyperthermia treatment studied so far, thus we believe that the therapeutic capabilities of AuAgNTrs herein demonstrated open the route to future cancer treatment applications.

## 4. Conclusions

In this paper we present an easy, reliable, and mild-conditions synthesis of AuAgNTrs stabilized with polyethylene glycol (PEG). The adjuvant role of silver led to the obtainment of ultrasmall gold-silver alloy nanotriangles. The average edge length of the nanotriangles appears to be roughly 20 nm: to the best of our knowledge, these are the smallest gold nanotriangles reported in the literature so far. Detailed TEM analysis revealed an Au-Ag solid solution (Au_70_Ag_30_) with an FCC crystal structure. The cell viability assay confirmed good biocompatibility even at high tested concentrations (70 μg AuAg/mL). Furthermore, their optical properties allow efficient photothermal treatment with the NIR laser, which was demonstrated in vitro.

## Data Availability

The data presented in this study are available on request from the corresponding author.
